# Serial Clustering of Late-Onset Group B Streptococcal Infections in the Neonatal Unit: A Genomic Re-evaluation of Causality

**DOI:** 10.1093/cid/ciy174

**Published:** 2018-03-02

**Authors:** Elita Jauneikaite, Georgia Kapatai, Frances Davies, Ioana Gozar, Juliana Coelho, Kathleen B Bamford, Benedetto Simone, Lipi Begum, Shannon Katiyo, Bharat Patel, Peter Hoffman, Theresa Lamagni, Eimear T Brannigan, Alison H Holmes, Tokozani Kadhani, Tracey Galletly, Kate Martin, Hermione Lyall, Yimmy Chow, Sunit Godambe, Victoria Chalker, Shiranee Sriskandan

**Affiliations:** 1 Health Protection Research Unit in Healthcare Associated Infections and Antimicrobial Resistance, Imperial College London, United Kingdom; 2 National Infection Service, Public Health England, United Kingdom; 3 Imperial College Healthcare NHS Trust, United Kingdom; 4 London and South East Field Epidemiology Services, United Kingdom; 5 North West London Health Protection Team, Public Health England, United Kingdom

**Keywords:** *Streptococcus agalactiae*, whole-genome sequencing, roup B streptococcus, neonate, outbreak

## Abstract

**Background:**

Invasive Group B streptococcus (GBS) is a major cause of serious neonatal infection. Current strategies to reduce early-onset GBS disease have no impact on late-onset disease (LOD). Although GBS LOD is viewed as a sporadic event in the community, LOD arising within the neonatal intensive care unit (ICU) raises questions about mode of acquisition.

**Methods:**

Following a cluster of 4 GBS LOD cases, enhanced surveillance for all GBS LOD was undertaken over 2 years in the neonatal ICU supported by neonatal rectal screening. GBS isolates were serotyped and genome-sequenced.

**Results:**

Twelve late -onset invasive GBS episodes were identified (incidence 0.6/1000 live births). Genomic analysis revealed that 11/12 GBS isolates (92%) were linked to at least one other LOD isolate. Isolates from the first cluster were serotype V, resistant to macrolides and lincosamides, and sequencing confirmed isolates were indistinguishable, or distinguishable by only one SNP difference, from each other. Rectal carriage was rare. Prospective surveillance identified three further clusters of LOD due to serotypes Ia (3 cases), Ib (2 cases), and III (2 cases), that would not have been identified without surveillance and genome sequencing, leading to a re-evaluation of interventions required to prevent GBS LOD.

**Conclusion:**

Acquisition routes for LOD GBS in the neonatal ICU are poorly understood; cases may not necessarily be sporadic. Within this neonatal ICU, our data suggest that a single case of LOD GBS sepsis should be considered a potential nosocomial transmission event warranting prompt investigation, heightened infection prevention vigilance and action where required.

## Teaser

Genomic surveillance for 2 years revealed that 11 of 12 late-onset group B streptococcus (GBS) infections in one neonatal intensive care unit were linked to ≥1 other GBS case, suggesting that even isolated late-onset GBS cases represent probable nosocomial transmission.

*Streptococcus agalactiae* (group B streptococcus [GBS]) is a major causal agent of neonatal sepsis [1]. GBS early-onset disease (EOD) affects infants within the first 6 days of life and is largely attributed to vertical transmission and colonization at the time of birth, while GBS late-onset disease (LOD) develops between 7 days and 3 months of age, resulting from vertical and potentially horizontal acquisition [2, 3]. GBS is carried asymptomatically as part of the enteric and vaginal microbiota of 11–35% of healthy women worldwide [4–6]. Interventions to reduce the incidence of EOD have been developed, but in the absence of a vaccine, these have minimal to no impact on the incidence of LOD [2, 7]. In contrast to EOD, interventions to prevent LOD are poorly researched, because LOD cases are assumed to be largely sporadic. Although nosocomial outbreaks of GBS have been described elsewhere [3, 8], the frequency of such outbreaks is presumed to be low [3].

We describe a series of clusters of invasive GBS LOD in a neonatal intensive care unit (NICU) that were due to 4 distinct GBS serotypes over a 24-month period. The first outbreak was initially recognized due to simultaneous presentations of GBS bacteremia with isolates of identical antimicrobial resistance profiles, and was confirmed by whole-genome sequencing. Enhanced surveillance identified 8 more isolates from GBS LOD over the following 23 months. Unlike the first cluster, none of the subsequent LOD isolates were isolated on the same day as any other LOD isolate and therefore would normally have been considered sporadic. However, genomic analysis demonstrated that almost every LOD GBS isolate in the NICU was phylogenetically associated with another LOD GBS isolate, pointing to a common but undetermined source for each cluster.

## METHODS

### Patients and Setting

Neonates and infants were cared for in a level 3 NICU with 24 cot spaces. GBS case patients were defined as neonates or infants from whom GBS was isolated from a normally sterile site while they were in the NICU. Colonization was defined as the isolation of GBS from a nonsterile site without evidence of site-specific infection. All neonates admitted to the NICU had ear swab samples routinely obtained at birth and received antibiotics for the first 48 hours of life. Enhanced surveillance for neonatal GBS colonization was begun in the NICU at the times indicated, using weekly rectal swab samples in all neonates and nose swab samples for a brief trial period.

Infection control measures and environmental surveillance took place as soon as an outbreak was suspected. Case records of affected neonates were reviewed. Contacts with healthcare workers (HCWs) were assessed using routinely held rostering records. Consent was obtained to report the cases described; individual consent for analysis of anonymized bacterial isolates was not required.

### GBS Isolates and Genome Sequencing

Nonsterile site and sterile site samples were cultured on selective or nonselective blood agar, respectively; identification, serotyping, antimicrobial susceptibility testing, DNA extraction, and genome sequencing were conducted as described in the Supplementary Methods. Genomic analyses compared sequences with available contemporaneous GBS of the same or closest sequence type (ST) (Supplementary Table 1). Sequences were submitted to the European Nucleotide Archive, reference PRJEB18093.

## RESULTS

### Cluster 1

**Figure 1.**
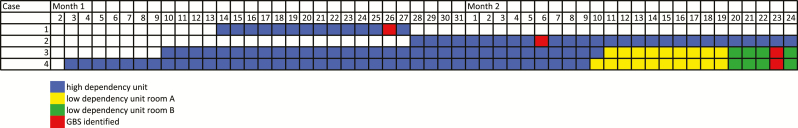
Cluster 1 timeline of serotype V group B streptococcus (GBS) late-onset disease (LOD) cases. Timeline shows admission to the neonatal intensive care unit (NICU), with onset of each case of GBS LOD (positive blood culture) represented by a single box in each case. Different areas of the NICU are indicated by different shades, and numbers on timeline represent dates in months 1 and 2.

In a 4-week period, 4 cases of LOD GBS bacteremia were detected in the NICU. Cases 1 and 2 arose 9 days apart within the high-dependency area, although the stays of the patients did not overlap (Figure 1). The first patient died 24 hours after onset of sepsis. Seventeen days after case 2, cases 3 and 4 arose within a 24-hour period in the low-dependency area; both patients had been in the NICU for a prolonged period (Figure 1). Cases 3 and 4 were linked in time and by cot space location, prompting recognition of an outbreak, underlined by the antimicrobial resistance phenotype. GBS isolates were resistant to tetracycline, macrolides, and lincosamides (Supplementary Table 2). An association with cases 1 and 2 was identified owing to identical antimicrobial resistance patterns, coupled with the unusually high frequency of LOD cases. All 4 GBS isolates were serotype V. During this 4-week period, there was a single episode of EOD due to GBS serotype Ia, which was therefore unrelated to the LOD cases.

The 4 cases of GBS serotype V LOD were characterized by gestational age, mode of delivery, and disease type (Table 1). At the time of birth, all 4 neonates were premature and required admission to the high-dependency NICU; all received empiric antimicrobials for suspected sepsis at the time of birth that were later discontinued in the absence of evidence for infection. Based on results of ear swab samples, there was no evidence of GBS colonization at the time of birth. None of the mothers were screened antenatally for GBS. At the time of onset of GBS LOD, all case patients had spent more than 7 days in the NICU (Figure 1), none were cannulated or intubated, and all were fed enterally.

**Table 1.** Case Patients in Group B Streptococcus Cluster 1 (Serotype V)

Patient Characteristics	Case 1	Case 2	Case 3	Case 4
Gestational complications	Breech TTS	Placenta previa; APH	Breech; *Candida* chorioamnionitis	Severe IUGR
Mode of delivery	ELSCS	SVD	SVD	ELSCS
Gestational age at birth, wk	27	24	26	29
Weight at birth, g	860	630	845	655
Results of ear swab sample culture at birth	Negative	Negative	*Candida albicans*	Negative
Antibiotics after birth (duration)	Penicillin-gentamicin (48 h)	Penicillin-gentamicin (48 h)	Penicillin-gentamicin (48 h); fluconazole (14 d)	Penicillin-gentamicin (48 h); tazocin-vancomycin (48 h); meropenem (5 d)
Gestational age at onset of GBS, d (site of GBS isolation)	12 (Blood)	9 (Blood)	44 (Blood)	51 (Blood)
Contact with GBS case patient	None known	Same bay as case patient 1, no overlap	Adjacent to case patient 4	Adjacent to case patient 3

Abbreviations: APH, antepartum hemorrhage; ELSCS, emergency lower segment cesarean section; GBS, group B streptococcus; IUGR, intrauterine growth retardation; SVD, spontaneous vaginal delivery; TTS, twin transfusion syndrome.

### GBS Colonization Screening

In the subsequent 3 months, weekly neonatal rectal swab sample screening was instituted in the NICU. GBS rectal colonization was not detected in any neonate or infant in the NICU. HCWs were not screened for GBS carriage, although none was identified as having shared contact with all 4 affected cases. Environmental sampling of equipment, cots, and surface areas failed to detect GBS in the NICU. After a 3-month enhanced surveillance period, during which no more cases of LOD or GBS colonization were detected, the outbreak was declared over, and screening was curtailed. Prospective enhanced surveillance for invasive LOD cases continued.

### Genomic Analysis of Serotype V Strains

Serotype V outbreak strains were multilocus sequence type ST1 with antibiotic resistance genes *tetM* and *ermB*. Genomic analysis indicated that all 4 outbreak strains were indistinguishable or distinguishable by only 1 single-nucleotide polymorphism (SNP). Comparison with serotype V reference genomes identified a lack of mobile genetic element RDF.1, previously described in invasive serotype V ST1 isolates [9] (Supplementary Figure 1*A*–*C*).

**Figure 2.**
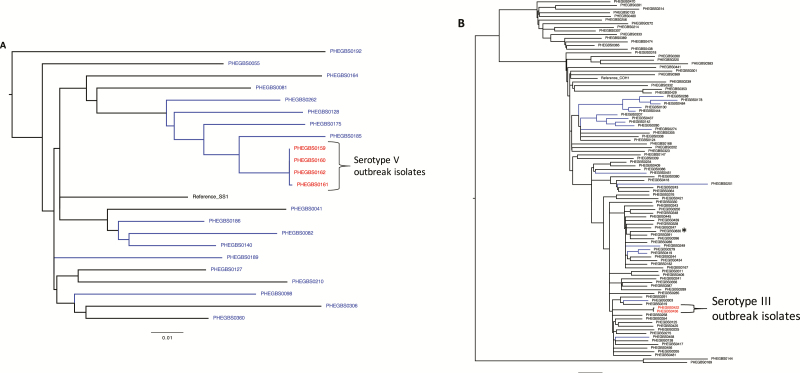

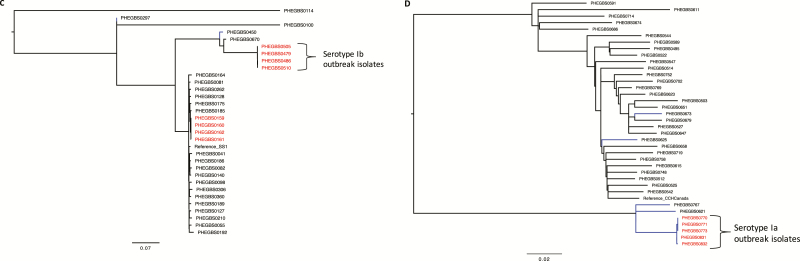
Phylogenetic analysis of outbreak cluster strains in the context of other UK isolates. Single-nucleotide polymorphism (SNP)–based approximate maximum likelihood phylogeny trees were constructed using FastTree (Supplementary Methods). Isolates with 0–2-SNPs differences were considered to share a common source; putative outbreaks are colored red. Scale bars indicate the nucleotide substitutions per site. Blue tree branches indicate isolates with tetracycline as well as macrolide/lincosamide resistance genes. Details for strains used in the analysis are provided in Supplementary Table 1. *A,* Cluster 1: Phylogeny tree based on 1104 core SNPs of 4 putative outbreak isolates and all 18 contemporaneous serotype V sequence type (ST) 1 isolates from the same year with reference sequence *S. agalactiae* SS1 (NZ_CP010867.1). There was 1-SNP difference between outbreak isolate PHEGBS0161 and the other 3 outbreak isolates, PHEGBS0159, PHEGBS0160, and PHEGBS0162. The next closest isolate PHEGBS0185 was 46–47 SNPs away from the outbreak isolates, and the most distant isolate PHEGBS0192 was 194–195 SNPs away. *B,* Cluster 2: Phylogeny tree based on 4844 core SNPs of 2 serotype III putative outbreak isolates, a single serotype III sporadic late-onset disease (LOD) isolate (*asterisk*), and 98 available whole-genome sequences of invasive serotype III ST17 from the same year with Refseq *S. agalactiae* COH1 (NZ_HG939456). There was 1-SNP difference between putative outbreak isolates PHEGBS0422 and PHEGBS036, whereas there were 91–92 SNPs between the outbreak isolates and the sporadic serotype III LOD isolate. The next closest isolates—PHEGBS0258, PHEGBS0319, and PHEGBS0354—were each 71–72 SNPs away from the outbreak isolates, and the most distant isolate PHEGBS0314 was 294–295 SNPs away. *C,* Cluster 3: Phylogeny tree based on 8862 core SNPs of serotype Ib ST139 isolates (n = 4) from putative outbreak and single-locus variants (ST1 and ST3) of serotypes Ib, II, V, and VI (n = 27) with ST1 reference sequence *S. agalactiae* SS1 (NZ_CP010867.1); the phylogeny tree includes all ST1 isolates shown in *A*, including cluster 1. Two invasive isolates from the putative 1b outbreak were identical, with no SNP difference. The rectal swab sample isolate PHEGBS0505 differed by 5 SNPs from blood isolates PHEGBS0510 and PHEGBS0486. The nearest contemporaneous isolate PHEGBS0670 was 761–762 SNPs away from the serotype Ib ST139 cluster isolates. *D,* Cluster 4: Phylogeny tree based on 1696 core SNPs of 37 serotype Ia ST23 isolates with Refseq *S. agalactiae* CCH210801006 (ERS337511). Blood and colonization isolates (n = 4) from the putative serotype Ia outbreak differed from each other by just 0–2 SNPs. The nearest contemporaneous group B streptococcus isolates, PHEGBS0621 and PHEGBS0767, differed from the putative outbreak isolates by 74–75 SNPs.

To provide context for the GBS sequences from cluster 1, sequences from 18 unrelated invasive GBS serotype V ST1 isolates were used in SNP analysis, obtained in the same year from adults and infants. This revealed interpositioning of adult and neonatal strains within the phylogeny tree (Figure 2) suggesting a shared, common reservoir, consistent with previous reports [10]. Thirteen of 22 UK ST1 GBS isolates carried *tetM* and macrolide resistance genes; of these, 8 that clustered together carried *ermB* (including the outbreak strains) (Figure 2A). Phylogenetic comparison of UK strains with serotype V ST1 strains from North America showed the same intermix of adult and neonatal strains and clustering of the *tetM*- and *ermB*-positive strains (Supplementary Figure 2).

### Prospective Enhanced Surveillance for GBS LOD

Recognition of the serotype V cluster prompted enhanced prospective surveillance for LOD in the NICU. Over the subsequent 2 years, 8 more GBS LOD isolates were identified.

#### Cluster 2

Nine months after onset of the first serotype V cluster, 2 GBS LOD bacteremia isolates were identified, and both were typed as serotype III. Weekly rectal screening of all neonates was resumed, but GBS colonization was not detected at that time. Genomic analysis revealed that cluster 2 consisted of ST17 serotype III GBS isolates that differed by a single SNP, indicating a very recent common ancestor (Figure 2B). The unique clustering of these 2 strains, when compared with contemporaneous serotype III ST17 strains from the United Kingdom, supported the likelihood of a common source.

#### Cluster 3

Ten months after the first cluster, GBS isolates from 2 more LOD bacteremias were identified 12 days apart and were typed as serotype Ib. In addition, a GBS rectal screening isolate from a third neonate, obtained 9 days before the first bacteremia, was serotype Ib, as was a nasal GBS isolate from a fourth neonate obtained during a trial period, 12 days after the first bacteremia. Genome sequencing showed that all 4 GBS serotype Ib isolates (2 invasive and 2 colonization) were from the ST139 lineage and clustered together with no SNP difference between the 2 invasive isolates, and with either 2- or 5-SNP differences between invasive and colonization isolates (Figure 2C). Contemporaneous isolates of serotype Ib ST139 were not available for comparison; therefore, single-locus variants of ST139, such as ST1 and ST3, were used in analysis (Supplementary Table 1). Analysis confirmed that the 4 serotype Ib ST139 isolates formed a unique cluster, consistent with transmission or a common source of infection (Figure 2C).

#### Cluster 4

Nearly 20 months after the first case in the first cluster, a fourth cluster of GBS LOD was identified. Three LOD bacteremia cases that arose over a 3-month period were due to GBS serotype Ia. Rectal colonization with GBS serotype Ia was observed in 1 bacteremic neonate (despite treatment and clearance from the blood) and in another routinely screened, nonbacteremic neonate. Genome sequencing confirmed that all 5 isolates differed from each other by only 1–2 SNPs and clustered together when compared with contemporaneous serotype Ia ST23 GBS strains (Figure 2D).

**Figure 3.**
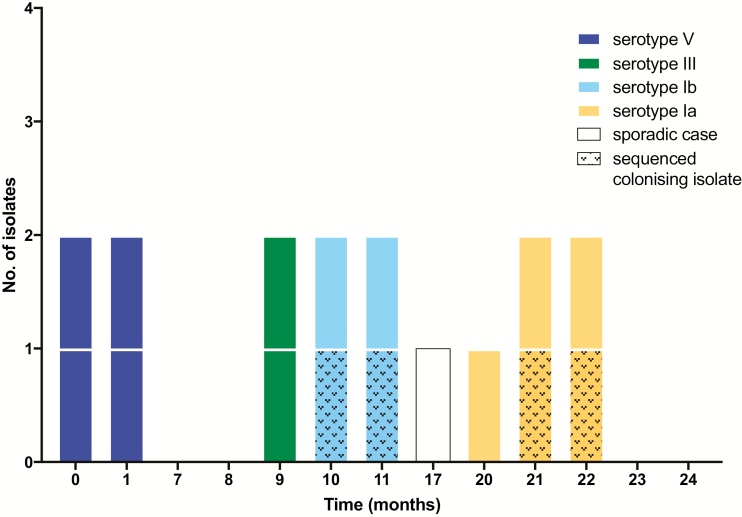
Timeline of all late-onset disease group B streptococcus (GBS) isolates and cluster-associated colonizing GBS isolates. Solid-shaded bars represent cluster-associated invasive isolates; stippled bars, cluster-associated colonization isolates. Different shades indicate GBS serotype.

During the 2-year enhanced surveillance period, only 1 LOD GBS case arose in which the isolate was phylogenetically unrelated to any other. This was a serotype III ST17 GBS strain isolated 16 months after the first cluster (Figure 3), and it was 91–92 SNPs different from the serotype III isolates that formed cluster 2 (Figure 2B). As such, it was deemed highly unlikely to have originated from a common source or to represent transmission from cluster 2.

### Review of Practice

Hand hygiene practice was reviewed and noted to be robust. However, breast pump hygiene was noted to be defective after the first cluster; swabbing of breast pumps yielded *Staphylococcus aureus* in 1 of 12 breast pumps tested. Additional infection control measures were implemented after the later clusters, including refined procedures for decontamination of neonatal equipment, continued weekly rectal carriage screening for each neonate, review of hand hygiene procedures, and new facilities and procedures for breast milk expression and storage, including single-use breast pumps. The number of cots in the NICU was reduced, and the area around each cot was increased. Prospective enhanced surveillance for GBS infections in the NICU is still ongoing.

## DISCUSSION

Four distinct clusters of invasive GBS LOD occurred in a single NICU over a 2-year period. These clusters involved all cases of LOD that arose during the same time period, apart from a single sporadic case, demonstrating the potential dominance of nosocomial transmission as a vehicle for LOD in this setting. The first outbreak of serotype V GBS LOD affecting 4 neonates was confirmed by means of whole-genome sequencing and highlighted the potential risk of horizontal transmission of GBS within the NICU. Importantly, recognition of the extent of the outbreak relied heavily on the antimicrobial resistance phenotype of the GBS isolates concerned and the coincidental presentation of the later 2 cases on the same day. Prospective surveillance of LOD cases identified likely transmission events on several later occasions in the NICU associated with different lineages of GBS, which resulted in clinically significant disease. It is likely that these transmission events would not have been recognized but for the earlier outbreak and subsequent enhanced surveillance, supported by genomic investigation.

Epidemiological investigation during the first outbreak was unable to identify a single common source, although genome sequencing demonstrated that all 4 GBS isolates formed a unique genetic cluster when compared to nonoutbreak strains of the same ST and serotype. GBS outbreaks are thought to be rare but have been reported elsewhere in the NICU setting [3, 8, 16], after discharge from hospital [11], and between adult patients [12], although, to our knowledge, whole-genome sequencing has not previously been used to confirm relatedness. Although the mode of transmission was unclear in these earlier reports, a potential breakdown of multiple hygiene practices has been identified as a likely cause [3, 8]. Indeed, older reports suggest that transmission may be more frequent in lower-dependency postnatal environments, affecting other patients and staff [13, 14].

EOD and LOD are most often caused by GBS serotypes III and Ia, with the highest risk of serotype III infections in LOD [1, 15]. Serotype V is a less common cause of neonatal disease in the United Kingdom but is widely recognized as a frequent cause of GBS disease in adults [1, 9]. Horizontal transmission of GBS LOD has been strongly associated with maternal carriage at the time of GBS onset; intrapartum antibiotics do not protect against LOD, indicating that later acquisition of, or even persistence of, GBS may underlie most cases [3]. Maternal carriage of GBS was not actively sought in the current study; screening of all mothers in the NICU was thought to be undesirable at the time of an outbreak, noting that antepartum GBS screening is not routinely undertaken in the United Kingdom. Although maternal colonization could account for index cases in clusters, it seemed unlikely to explain all subsequent cases which, taken together, pointed to single origins for each cluster.

HCWs were also not screened during the outbreak, because there were no common contacts, and there are no data to guide the type of sample or action required should HCWs be found to be colonized. GBS is part of the normal gut and genital microbiota, and though GBS nasopharyngeal colonization has been reported, it is poorly understood [13, 17]; no clearance protocol has been shown to be reliable. Therefore, reinforcement of hand and environmental hygiene seemed the most suitable option to manage the outbreaks. The findings of this study underline the potential value of a widened genomic study of the GBS reservoir within the NICU that includes staff, parents, and neonates.

Although GBS contamination of the environment could not be discounted or proved, it was evident that breast pump decontamination was suboptimal. Thus, improved cleaning and disinfection practices were implemented, with an emphasis on single-patient-use equipment [18]. Breast milk contamination has been linked to LOD [16, 19, 20], although this linkage has not been subject to systematic study in the NICU setting. It is possible that low-grade contamination of equipment related to feeding led to neonatal exposure. Although GBS can be found as a normal commensal in the gut microbiota of healthy infants [21], it was rarely identified by rectal screening of NICU patients in our study; of 518 admissions in 1 year, GBS rectal carriage was identified in just 10 individual patients (1.9%), including 1 with GBS late-onset bacteremia. The low rate may be because a greater proportion of NICU patients are delivered by cesarean section and are of very low birth weight, resulting in gut microbiota distinct from that in healthy infants [22]. Little is known about the timing of GBS colonization among preterm low-birth-weight infants in the NICU and the impact of enteral feeding of maternal or donor breast milk. In the current study, among the few neonates who did acquire rectal GBS, the median time to acquisition was 24.5 days after birth (range, 13–68 days).

Antimicrobial resistance played a large part in identification of the first cluster, as reported in other bacterial outbreaks, for example, with methicillin-resistant Staphylococcus aureus [23]. Resistance to tetracycline, erythromycin, and clindamycin was confirmed by the presence of *tetM* and *ermB* genes in all 4 serotype V GBS outbreak isolates in the first cluster. Tetracycline resistance has been reported in 78% of GBS isolates in the United Kingdom [24] and it has been suggested that acquisition of *tetM* was a driving force for emergence of GBS as a human pathogen in the 1960s [25]. Erythromycin and clindamycin resistance conferred by *ermB* is increasingly observed among serotype V GBS [26] and is a concern because erythromycin has been used as prophylaxis for preterm premature rupture of membranes, and clindamycin has been used for peripartum sepsis or intrapartum antibiotic prophylaxis in those allergic to penicillin or in severe sepsis. The high prevalence of macrolide and lincosamide resistance determinants supports revision of these protocols (Supplementary Table 1).

Genomic outbreak investigation requires large-scale databases, which include longitudinally collected data from both clinical and carriage isolates to provide context for clusters, and to allow more certainty regarding transmission events. Such data were invaluable in analyzing the outbreaks described herein. Outbreak isolates were identical or differed from each other by 1–2 SNP, whereas the closest, unrelated isolates from the same year differed by >45 SNPs, providing useful context for future investigations. Phylogenetic analysis indicated that adult and newborn GBS isolates intermixed, confirming that adult and neonatal strains originate from the same genetic pool [10] albeit that adult GBS isolates tend to be more diverse than infant strains [1].

Routine antepartum screening for GBS and intravenous antibiotic prophylaxis has resulted in reduced EOD in North America, but no reduction was observed for LOD [27]. Despite a wealth of guidance [28] to prevent GBS EOD, we are unaware of any interventions to specifically prevent GBS LOD or nosocomial transmission. The rate of LOD GBS in the period studied was 0.6/1000 live births. We considered the possibility that our unit may be an outlier with regard to risk of infection, but the Vermont Oxford Network risk-adjusted standardized mortality ratio for late-onset bacterial infection was below average in this period, and there was no consistent increase in other bacterial infections.

The outbreak and subsequent demonstration of further horizontal transmission events in the NICU has highlighted a lack of understanding of the modes of GBS transmission within the hospital environment, specifically the possible role of HCWs, mothers, the environment, feeding and lactation equipment, and the prospect that a significant proportion of LOD arising in hospital may be nosocomially transmitted. These are all areas of future research focus. Given that GBS is an infrequent finding in the enteric microbiota of neonates in the NICU setting, we believe that a single case of LOD arising in the NICU should be considered a potential sentinel case of a future outbreak prompting enhanced retrospective and prospective surveillance for more cases and investigation of preventable sources of transmission. Whether similar events occur in other NICU settings will require prospective surveillance similar to that implemented in our study.

## Supplementary Data

Supplementary materials are available at *Clinical Infectious Diseases* online. Consisting of data provided by the authors to benefit the reader, the posted materials are not copyedited and are the sole responsibility of the authors, so questions or comments should be addressed to the corresponding author.

Supplementary Material

## Notes

***Author contributions.*** All authors collected clinical and epidemiological data on Group B streptococcus Late-onset disease, infection control measures, and environmental surveillance that took place. V. C. and S. S. coordinated the microbiological and genomic analysis. E. J. and G. K. analyzed whole-genome sequencing data. J. C. collected epidemiology data for contemporaneous strains, carried out serotyping and antimicrobial susceptibility testing. E. J., T. L., and S. S. analyzed the data and prepared tables and figures. E. J. and S. S. wrote the first draft of manuscript. E. J., F. D., T. L., V. C., and S. S. contributed to further drafts, and all authors reviewed and edited the final manuscript.

***Acknowledgments.*** We thank the parents and all nursing, clinical, and Microbiology laboratory staff for support and Paul Heath and Jane Plumb for helpful comments in preparation of this manuscript. We acknowledge the Genomic Services and Development Unit (Catherine Arnold, Public Health England [PHE]) for next-generation sequencing, and the Respiratory and Vaccine Preventable Bacteria Reference Unit, PHE, for serotyping and DNA extractions. Thanks are also due to microbiologists around the United Kingdom for submission of contemporaneous isolates used in this analysis and to the British Paediatric Surveillance Unit (BPSU) study (Surveillance of Group B streptococcal disease in infants <90 days of age, a collaboration by the BPSU, PHE, and St George’s, University of London) for DNA extractions and serotyping of these isolates.

***Disclaimer.*** The funding sources had no role in study design, data collection, analysis, or decision to submit the manuscript for publication. The views expressed are those of the author(s) and not necessarily those of the National Health Service, the National Institute for Health Research (NIHR), the UK Department of Health, or Public Health England.

***Financial support.*** This work was supported by the NIHR Health Protection Research Unit in Healthcare Associated Infections and Antimicrobial Resistance at Imperial College London, in partnership with PHE, the UK Clinical Research Collaboration (National Centre for Infection Prevention & Management), and the Respiratory and Vaccine Preventable Bacteria Reference Unit at PHE, and also by the NIHR Biomedical Research Centre at Imperial College.

***Potential conflicts of interest.*** All authors: No reported conflicts. All authors have submitted the ICMJE Form for Disclosure of Potential Conflicts of Interest. Conflicts that the editors consider relevant to the content of the manuscript have been disclosed.
