# Supplementary material for: Serial Clustering of Late-Onset Group B Streptococcal Infections in the Neonatal Unit: A Genomic Re-evaluation of Causality
Source: Clin Infect Dis. 2018 Mar 2;67(6):854–60. doi: 10.1093/cid/ciy174 (PMC6117446; doi:10.1093/cid/ciy174)
Supplement: Supplementary Material [file ciy174_suppl_supplementary_material.doc]

**Supplementary material**

**Supplementary methods**

**GBS isolates.** Rectal and other non-sterile site swabs were cultured on Columbia blood agar with 8mg/l aztreonam (Oxoid, UK), blood cultures were cultured on Columbia blood agar (Oxoid, UK). GBS were identified using MALDITOF (Bruker, UK) and confirmed with streptococcal grouping kit (Oxoid, UK) if MALDITOF results were inconclusive . Susceptibility to anibiotics penicillin, erythromycin, tetracycline, clindamycin, teicoplanin and vancomycin (Suppl. Table 2) were determined via agar dilution. For erythromycin and clindamycin, a 0.06 to 128 *µ*g/mL range of antibiotic concentrations were tested.  Iso‑Sensitest agar containing 5% horse blood (Oxoid, Thermo Scientific, UK) was inoculated with 1.5x10^8^CFU/spot of sample using a multipoint inoculator and incubated aerobically overnight at 37°C. British Society of Antimicrobial Chemotherapy (BSAC) criteria were used to determine whether isolates were resistant or susceptible to erythromycin (susceptibility and resistance, ≤0.25*µ*g/mL and >0.5 *µ*g/mL) and clindamycin (susceptibility, ≤0.5 *µ*g/mL) [1]. Neonatal GBS isolates were serotyped using latex agglutination (Staten Serum Insitute, Denmark).

**Genome sequencing.** DNA was prepared using Wizard Genomic DNA Purification kit ( Promega, USA). Nextera XT DNA Library Preparation kit (Illumina, USA) was used to prepare multiplexed DNA sequencing libraries. Whole genome sequencing was undertaken using Illumina HiSeq 2500 System (Illumina, US) and 2 x 100bp paired-end mode. Reads were submitted as PHE Pathogens BioProject PRJEB18093 at ENA - http://www.ebi.ac.uk/ena/data/view/ PRJEB18093 (Supplementary table 1).

**Genomic analyses.** Reads were assembled using SPAdes[2]. MOST[3] was used to call multi-locus sequence type (MLST) and SRST2[4] was used to identify presence (minimal gene coverage of 90%) of resistance genes conferring resistance to tetracycline, macrolides and lincosamides (based on resistance patterns shown by outbreak isolates) using antibiotic resistance allele databases ARGannot and ResFinder as per Metcalf *et al* 2017 [5]. Single nucleotide polymorphisms (SNP) were called using Snippy v2.5/Freebayes v0.9.21-7 (<https://github.com/tseemann/snippy)> with default settings. Approximate maximum likelihood phylogenetic trees were prepared from core SNPs using FastTree [6] and visualised using FigTree (http://tree.bio.ed.ac.uk/software/figtree/).

**Selection of contemporaneous isolates.** Sterile site isolates from both infants and adults are routinely submitted to the Public Health England reference laboratory as part of national surveillance. Available GBS whole genome sequences from invasive isolates submitted to PHE between January 2014 and December 2015 were used to select contemporaneous isolates for phylogenetic analysis of each cluster. GBS isolates were selected from invasive isolates submitted to the reference laboratory in the same year as the cluster involved, and matched by MLST and, where possible, serotype as follows: for **cluster 1,** Serotype V outbreak isolates were compared with 18 other, non-outbreak invasive GBS isolates of the same serotype and ST (serotype V, ST1) from England, from both neonates and adults; additionally, serotype V cluster isolates were compared to 40 published serotype V, ST1 invasive GBS sequences from North American adults [7]. Serotype V isolate genome sequences were also compared with available reference sequences SS1 (NZ_CP010867.1, ST1 invasive disease isolate from an adult); 2603V/R (NC_004116.1, ST110 invasive disease isolate from an adult) and 09mas018883 (NC_021485.1, ST1 isolate from bovine mastitis) to identify the closest refseq to be used in phylogenetic analysis. Refseq SS1 (NZ_CP010867.1) was used for SNPs calling and phylogenetic analysis. For **cluster 2**, ninety-eight contemporaneous isolates of serotype III, ST17, and refseq COH1 (NZ_HG939456, serotype III, ST17) were used to investigate the serotype III cluster (2 cases) and one sporadic case of serotype III. For **cluster** **3**, (serotype Ib , ST139 cluster), no other contemporaneous serotype Ib ST139 isolates were available for comparison, therefore, available genomic sequences of single locus variants of ST139 (ST1 and ST3) from the same year were used instead, regardless of serotype. This included two serotype Ib /ST1 isolates; 22 serotype V /ST1 isolates (as used in cluster 1 analysis including the serotype V outbreak isolates); one serotype VI /ST1 isolate; and two serotype II/ST3 isolates. For **cluster 4** (serotype Ia, ST23), thirty-six contemporaneous isolates of serotype Ia, ST23 and refseq CCH210801006 (ERS337511, serotype Ia, ST23) were used to investigate the cluster isolates.

**References for supplementary material**

1. British Society for Antimicrobial Chemotherapy. Standing committee on susceptibility testing. **2015**. Version 14.0.

2. Bankevich A, Nurk S, Antipov D, et al. SPAdes: a new genome assembly algorithm and its applications to single-cell sequencing. J Comput Biol **2012**; 19(5): 455-77.

3. Tewolde R, Dallman, T., Schaefer, U., Sheppard, C.L., Ashton, P., Pichon, B., Ellington, M., Swift, C., Green, J. and Underwood, A., . MOST: A modified MLST typing tool based on short read sequencing. PeerJ Preprints **2016**; (No. e1971v1).

4. Inouye M, Dashnow H, Raven LA, et al. SRST2: Rapid genomic surveillance for public health and hospital microbiology labs. Genome Medicine **2014**; 6(11): 90.

5. Metcalf BJ, Chochua S, Gertz RE, Jr., et al. Short-read whole genome sequencing for determination of antimicrobial resistance mechanisms and capsular serotypes of current invasive Streptococcus agalactiae recovered in the USA. Clinical Microbiology and Infectious Diseases **2017**; 23(8): 574 e7- e14.

6. Price MN, Dehal PS, Arkin AP. FastTree: computing large minimum evolution trees with profiles instead of a distance matrix. Mol Biol Evol **2009**; 26(7): 1641-50.

7. Flores AR, Galloway-Pena J, Sahasrabhojane P, et al. Sequence type 1 group B Streptococcus, an emerging cause of invasive disease in adults, evolves by small genetic changes. Proc Natl Acad Sci U S A **2015**; 112(20): 6431-6.

8. Alikhan NF, Petty NK, Ben Zakour NL, Beatson SA. BLAST Ring Image Generator (BRIG): simple prokaryote genome comparisons. BMC Genomics **2011**; 12: 402.

**Supplementary Tables**

**Suppl. Table 1. Summary of the GBS LOD cluster and contemporaneous UK isolates used in the analysis.** Isolates shaded in grey are from identified clusters. The GBS LOD isolate identified from prospective enhanced surveillance, but not part of any identified cluster, is in bold font. x indicates that the particular resistance determinants were not identified in the whole genome sequence.

| **Sample ID** | **Serotype** | **ST** | | | **Specimen** | **Age group** | **Age (yrs)** | **Year of isolation** | **Antibiotic resistance genes** | | | | | | | **SRA number** |
| --- | --- | --- | --- | --- | --- | --- | --- | --- | --- | --- | --- | --- | --- | --- | --- | --- |
|  |  |  |  |  |  |  |  |  | **Tet** | | | | | **MLS** | |  |
| **Serotype V** | | | | | | | | | | | | | | | | |
| PHEGBS0159 | V | ST1 | | | blood | infant | 0 | 2014 | *tetM* | | *ermB* | | | | | ERR1741603 |
| PHEGBS0160 | V | ST1 | | | blood | infant | 0 | 2014 | *tetM* | | *ermB* | | | | | ERR1742046 |
| PHEGBS0161 | V | ST1 | | | blood | infant | 0 | 2014 | *tetM* | | *ermB* | | | | | ERR1742093 |
| PHEGBS0162 | V | ST1 | | | blood | infant | 0 | 2014 | *tetM* | | *ermB* | | | | | ERR1741454 |
| PHEGBS0041 | V | ST1 | | | blood | adult | 74 | 2014 | *tetM* | | x | | | | | ERR1742042 |
| PHEGBS0055 | V | ST1 | | | blood | infant | 0 | 2014 | *tetM* | | x | | | | | ERR1741681 |
| PHEGBS0081 | V | ST1 | | | blood | adult | 86 | 2014 | *tetM* | | x | | | | | ERR1741497 |
| PHEGBS0082 | V | ST1 | | | blood | adult | 92 | 2014 | *tetM* | | *ermA* | | | | | ERR1741501 |
| PHEGBS0098 | V | ST1 | | | blood | adult | 23 | 2014 | *tetM* | | *ermA* | | | | | ERR1741573 |
| PHEGBS0127 | V | ST1 | | | blood | adult | 86 | 2014 | *tetM* | | x | | | | | ERR1741367 |
| PHEGBS0128 | V | ST1 | | | blood | adult | 80 | 2014 | *tetM* | | *ermB* | | | | | ERR1742140 |
| PHEGBS0140 | V | ST1 | | | blood | infant | 0 | 2014 | *tetM* | | *ermA* | | | | | ERR1741418 |
| PHEGBS0164 | V | ST1 | | | blood | adult | 59 | 2014 | *tetM* | | x | | | | | ERR1741749 |
| PHEGBS0175 | V | ST1 | | | unknown | infant | 0 | 2014 | *tetM* | | *ermB* | | | | | ERR1741838 |
| PHEGBS0185 | V | ST1 | | | blood | infant | 0 | 2014 | *tetM* | | *ermB* | | | | | ERR1741495 |
| PHEGBS0186 | V | ST1 | | | CSF | infant | 0 | 2014 | *tetM* | | *ermA* | | | | | ERR1741739 |
| PHEGBS0189 | V | ST1 | | | blood | infant | 0 | 2014 | *tetM* | | *mefA/msrD* | | | | | ERR1741991 |
| PHEGBS0192 | V | | ST1 | | blood | infant | 0 | 2014 | *tetM* | | | x | | | ERR1741359 | |
| PHEGBS0210 | V | | ST1 | | blood | infant | 0 | 2014 | *tetM* | | | x | | | ERR1741846 | |
| PHEGBS0262 | V | | ST1 | | blood | infant | 0 | 2014 | *tetM, tetO* | | | *ermB* | | | ERR1742083 | |
| PHEGBS0306 | V | | ST1 | | blood | adult | 27 | 2014 | *tetM* | | | x | | | ERR1741485 | |
| PHEGBS0360 | V | | ST1 | | blood | adult | 78 | 2014 | *tetM* | | | x | | | ERR1742032 | |
| **Serotype III** | | | | | | | | | | | | | | | | |
| PHEGBS0422 | III | | ST17 | | blood | infant | 0 | 2014 | *tetM* | | | | x | | | ERR1741522 |
| PHEGBS0436 | III | | ST17 | | blood | infant | 0 | 2014 | *tetM* | | | | x | | | ERR1742010 |
| **PHEGBS0830** | **III** | | **ST17** | | **blood** | **infant** | **0** | **2014** | ***tetM*** | | | | **x** | | | **ERR1924355** |
| PHEGBS0124 | III | | ST17 | | blood | infant | 0 | 2014 | x | | | | x | | | ERR1741386 |
| PHEGBS0125 | III | | ST17 | | blood | infant | 0 | 2014 | *tetM* | | | | x | | | ERR1741661 |
| PHEGBS0130 | III | | ST17 | | blood | infant | 0 | 2014 | *tetO* | | | | *ermB* | | | ERR1741468 |
| PHEGBS0133 | III | | ST17 | | CSF | infant | 0 | 2014 | *tetM* | | | | x | | | ERR1741601 |
| PHEGBS0138 | III | | ST17 | | blood | infant | 0 | 2014 | *tetM* | | | | x | | | ERR1741686 |
| PHEGBS0142 | III | | ST17 | | blood | infant | 0 | 2014 | *tetO* | | | | *ermB, lnuB* | | | ERR1741506 |
| PHEGBS0144 | III | | ST17 | | blood | adult | 28 | 2014 | *tetO* | | | | x | | | ERR1741588 |
| PHEGBS0147 | III | | ST17 | | blood | infant | 0 | 2014 | *tetM* | | | | x | | | ERR1742073 |
| PHEGBS0167 | III | | ST17 | | blood | infant | 0 | 2014 | *tetM* | | | | x | | | ERR1741977 |
| PHEGBS0168 | III | | ST17 | | blood | infant | 0 | 2014 | *tetM* | | | | x | | | ERR1741840 |
| PHEGBS0169 | III | | ST17 | | blood | infant | 0 | 2014 | *tetM* | | | | x | | | ERR1741558 |
| PHEGBS0178 | III | | ST17 | | blood | infant | 0 | 2014 | *tetO* | | | | *ermB, mefA/msrD* | | | ERR1741405 |
| PHEGBS0182 | III | | ST17 | | blood | infant | 0 | 2014 | *tetM* | | | | x | | | ERR1741489 |
| PHEGBS0201 | III | | ST17 | | blood | infant | 0 | 2014 | *tetM* | | | | *mefA/msrD* | | | ERR1741528 |
| PHEGBS0202 | III | | ST17 | | blood | infant | 0 | 2014 | x | | | | x | | | ERR1742113 |
| PHEGBS0207 | III | | ST17 | | blood | adult | 85 | 2014 | *tetO* | | | | *ermB* | | | ERR1741997 |
| PHEGBS0214 | III | | ST17 | | blood | adult | 59 | 2014 | *tetM* | | | | x | | | ERR1741810 |
| PHEGBS0220 | III | | ST17 | | blood | adult | 49 | 2014 | *tetM* | | | | x | | | ERR1741672 |
| PHEGBS0225 | III | | ST17 | | blood | infant | 0 | 2014 | *tetM* | | | | x | | | ERR1741440 |
| PHEGBS0234 | III | | ST17 | | blood | infant | 0 | 2014 | *tetM* | | | | x | | | ERR1741760 |
| PHEGBS0235 | III | | ST17 | | blood | infant | 0 | 2014 | *tetM* | | | | x | | | ERR1741417 |
| PHEGBS0239 | III | | ST17 | | blood | infant | 0 | 2014 | *tetM* | | | | x | | | ERR1741693 |
| PHEGBS0241 | III | | ST17 | | blood | infant | 0 | 2014 | *tetM* | | | | x | | | ERR1741732 |
| PHEGBS0243 | III | | ST17 | | blood | adult | 33 | 2014 | *tetM* | | | | x | | | ERR1741633 |
| PHEGBS0244 | III | | ST17 | | blood | infant | 0 | 2014 | *tetM* | | | | x | | | ERR1742105 |
| PHEGBS0247 | III | | ST17 | | blood | infant | 0 | 2014 | *tetM* | | | | x | | | ERR1742128 |
| PHEGBS0249 | III | | ST17 | | blood | infant | 0 | 2014 | *tetM, tetO* | | | | *ermB* | | | ERR1741370 |
| PHEGBS0250 | III | | ST17 | | blood | infant | 0 | 2014 | *tetM* | | | | x | | | ERR1741783 |
| PHEGBS0256 | III | | ST17 | | CSF | infant | 0 | 2014 | *tetM* | | | | x | | | ERR1741404 |
| PHEGBS0258 | III | | ST17 | | blood | infant | 0 | 2014 | *tetM* | | | | x | | | ERR1741578 |
| PHEGBS0260 | III | | ST17 | | blood | infant | 0 | 2014 | *tetM* | | | | x | | | ERR1741715 |
| PHEGBS0272 | III | | ST17 | | blood | infant | 0 | 2014 | *tetM* | | | | x | | | ERR1741358 |
| PHEGBS0274 | III | | ST17 | | CSF | infant | 0 | 2014 | *tetM, tetL* | | | | *ermX* | | | ERR1741376 |
| PHEGBS0275 | III | | ST17 | | blood | adult | 31 | 2014 | *tetM* | | | | x | | | ERR1742034 |
| PHEGBS0279 | III | | ST17 | | blood | infant | 0 | 2014 | *tetM, tetO* | | | | *ermB* | | | ERR1741663 |
| PHEGBS0280 | III | | ST17 | | blood | infant | 0 | 2014 | *tetO* | | | | *ermB, lnuB* | | | ERR1741682 |
| PHEGBS0281 | III | | ST17 | | blood | infant | 0 | 2014 | *tetM* | | | | x | | | ERR1741413 |
| PHEGBS0285 | III | | ST17 | | blood | infant | 0 | 2014 | *tetM* | | | | x | | | ERR1741855 |
| PHEGBS0286 | III | | ST17 | | blood | adult | 39 | 2014 | *tetM* | | | | x | | | ERR1741880 |
| PHEGBS0288 | III | | ST17 | | blood | adult | 88 | 2014 | *tetM* | | | | *ermB, mefA/msrD* | | | ERR1741473 |
| PHEGBS0289 | III | | ST17 | | blood | infant | 0 | 2014 | *tetM* | | | | x | | | ERR1741685 |
| PHEGBS0291 | III | | ST17 | | blood | infant | 0 | 2014 | *tetM* | | | | x | | | ERR1741704 |
| PHEGBS0301 | III | | ST17 | | blood | infant | 0 | 2014 | *tetM* | | | | x | | | ERR1741767 |
| PHEGBS0303 | III | | ST17 | | blood | infant | 0 | 2014 | *tetM* | | | | *ermT* | | | ERR1741491 |
| PHEGBS0305 | III | | ST17 | | blood | infant | 0 | 2014 | *tetM* | | | | x | | | ERR1741978 |
| PHEGBS0307 | III | | ST17 | | blood | infant | 0 | 2014 | x | | | | x | | | ERR1741953 |
| PHEGBS0311 | III | | ST17 | | blood | infant | 0 | 2014 | *tetM* | | | | x | | | ERR1741636 |
| PHEGBS0314 | III | | ST17 | | blood | unknown | unknown | 2014 | | *tetM* | | | x | | | ERR1742071 |
| PHEGBS0318 | III | | ST17 | | blood | adult | 89 | 2014 | | *tetM* | | | x | | | ERR1741792 |
| PHEGBS0319 | III | | ST17 | | blood | infant | 0 | 2014 | | *tetM* | | | x | | | ERR1741945 |
| PHEGBS0323 | III | | ST17 | | blood | infant | 0 | 2014 | | *tetM* | | | x | | | ERR1741384 |
| PHEGBS0328 | III | | ST17 | | blood | infant | 0 | 2014 | | *tetM* | | | x | | | ERR1741798 |
| PHEGBS0330 | III | | ST17 | | CSF | infant | 0 | 2014 | | *tetM* | | | x | | | ERR1741762 |
| PHEGBS0332 | III | | ST17 | | blood | infant | 0 | 2014 | | *tetM* | | | x | | | ERR1742004 |
| PHEGBS0333 | III | | ST17 | | blood | infant | 0 | 2014 | | *tetM* | | | x | | | ERR1741699 |
| PHEGBS0338 | III | | ST17 | | blood | infant | 0 | 2014 | | *tetM, tetL* | | | x | | | ERR1741598 |
| PHEGBS0339 | III | | ST17 | | blood | infant | 0 | 2014 | | *tetM* | | | x | | | ERR1741365 |
| PHEGBS0343 | III | | ST17 | | blood | infant | 0 | 2014 | | *tetM* | | | x | | | ERR1741667 |
| PHEGBS0348 | III | | ST17 | | blood | infant | 0 | 2014 | | *tetM* | | | x | | | ERR1741592 |
| PHEGBS0353 | III | | ST17 | | blood | infant | 0 | 2014 | | *tetM* | | | x | | | ERR1741836 |
| PHEGBS0354 | III | | ST17 | | blood | infant | 0 | 2014 | | *tetM* | | | x | | | ERR1741745 |
| PHEGBS0355 | III | | ST17 | | blood | adult | 24 | 2014 | | *tetM* | | | x | | | ERR1741387 |
| PHEGBS0356 | III | | ST17 | | blood | infant | 0 | 2014 | | *tetM* | | | x | | | ERR1741527 |
| PHEGBS0364 | III | | ST17 | | CSF | infant | 0 | 2014 | | *tetM* | | | x | | | ERR1742126 |
| PHEGBS0365 | III | | ST17 | | blood | infant | 0 | 2014 | | *tetM* | | | x | | | ERR1741569 |
| PHEGBS0369 | III | | ST17 | | CSF | infant | 0 | 2014 | | *tetM* | | | x | | | ERR1741552 |
| PHEGBS0376 | III | | ST17 | | blood | infant | 0 | 2014 | | *tetM* | | | x | | | ERR1741790 |
| PHEGBS0380 | III | | ST17 | | blood | adult | 30 | 2014 | | *tetM* | | | x | | | ERR1741867 |
| PHEGBS0383 | III | | ST17 | | blood | adult | 27 | 2014 | | *tetM* | | | x | | | ERR1741449 |
| PHEGBS0386 | III | | ST17 | | CSF | infant | 0 | 2014 | | *tetM* | | | x | | | ERR1741976 |
| PHEGBS0387 | III | | ST17 | | blood | unknown | unknown | 2014 | | *tetM* | | | x | | | ERR1741551 |
| PHEGBS0389 | III | | ST17 | | blood | adult | 20 | 2014 | | *tetM* | | | x | | | ERR1741939 |
| PHEGBS0391 | III | | ST17 | | blood | infant | 0 | 2014 | | *tetM* | | | x | | | ERR1741471 |
| PHEGBS0396 | III | | ST17 | | blood | infant | 0 | 2014 | | *tetM* | | | x | | | ERR1741738 |
| PHEGBS0406 | III | | ST17 | | blood | infant | 0 | 2014 | | *tetM* | | | x | | | ERR1741451 |
| PHEGBS0409 | III | | ST17 | | blood | infant | 0 | 2014 | | *tetM* | | | x | | | ERR1741881 |
| PHEGBS0417 | III | | ST17 | | blood | infant | 0 | 2014 | | *tetM* | | | x | | | ERR1741381 |
| PHEGBS0418 | III | | ST17 | | blood | infant | 0 | 2014 | | *tetM* | | | x | | | ERR1741544 |
| PHEGBS0419 | III | | ST17 | | blood | infant | 0 | 2014 | | *tetM, tetO* | | | *ermB* | | | ERR1741878 |
| PHEGBS0421 | III | | ST17 | | blood | infant | 0 | 2014 | | *tetM* | | | x | | | ERR1741395 |
| PHEGBS0422 | III | | ST17 | | blood | infant | 0 | 2014 | | *tetM* | | | x | | | ERR1741522 |
| PHEGBS0425 | III | | ST17 | | blood | infant | 0 | 2014 | | *tetM* | | | x | | | ERR1742097 |
| PHEGBS0429 | III | | ST17 | | blood | adult | 92 | 2014 | | *tetM* | | | x | | | ERR1741515 |
| PHEGBS0434 | III | | ST17 | | blood | infant | 0 | 2014 | | *tetM* | | | x | | | ERR1741357 |
| PHEGBS0437 | III | | ST17 | | blood | infant | 0 | 2014 | | *tetO* | | | *ermB* | | | ERR1741998 |
| PHEGBS0438 | III | | ST17 | | blood | infant | 0 | 2014 | | *tetM* | | | x | | | ERR1741560 |
| PHEGBS0439 | III | | ST17 | | blood | infant | 0 | 2014 | | *tetM* | | | x | | | ERR1741826 |
| PHEGBS0441 | III | | ST17 | | blood | infant | 0 | 2014 | | *tetM* | | | x | | | ERR1741530 |
| PHEGBS0444 | III | | ST17 | | blood | infant | 0 | 2014 | | *tetO* | | | *ermB* | | | ERR1741929 |
| PHEGBS0445 | III | | ST17 | | blood | infant | 0 | 2014 | | *tetM* | | | x | | | ERR1741414 |
| PHEGBS0451 | III | | ST17 | | CSF | infant | 0 | 2014 | | *tetM* | | | *emrB* | | | ERR1741403 |
| PHEGBS0456 | III | | ST17 | | CSF | infant | 0 | 2014 | | *tetM* | | | x | | | ERR1741439 |
| PHEGBS0458 | III | | ST17 | | blood | infant | 0 | 2014 | | *tetM* | | | *ermB* | | | ERR1741873 |
| PHEGBS0470 | III | | ST17 | | blood | infant | 0 | 2014 | | *tetM* | | | x | | | ERR1741676 |
| PHEGBS0474 | III | | ST17 | | CSF | infant | 0 | 2014 | | *tetM* | | | x | | | ERR1741809 |
| PHEGBS0480 | III | | ST17 | | blood | adult | 25 | 2014 | | *tetM* | | | x | | | ERR1741862 |
| PHEGBS0481 | III | | ST17 | | blood | infant | 0 | 2014 | | *tetM* | | | x | | | ERR1741854 |
| PHEGBS0484 | III | | ST17 | | blood | infant | 0 | 2014 | | *tetO* | | | *ermB* | | | ERR1742079 |
| **Serotype Ib** | | | | | | | | | | | | | | | | |
| PHEGBS0479 | Ib | | | ST139 | nose swab | infant | 0 | 2014 | | *tetM* | | | x | | | ERR1742007 |
| PHEGBS0486 | Ib | | | ST139 | blood | infant | 0 | 2014 | | *tetM* | | | x | | | ERR1741994 |
| PHEGBS0505 | Ib | | | ST139 | rectal swab | infant | 0 | 2014 | | *tetM* | | | x | | | ERR1741868 |
| PHEGBS0510 | Ib | | | ST139 | blood | infant | 0 | 2014 | | *tetM* | | | x | | | ERR1741777 |
| PHEGBS0450 | Ib | | | ST1 | blood | adult | 74 | 2014 | | *tetM* | | | *emrB* | | | ERR1741493 |
| PHEGBS0670 | Ib | | | ST1 | blood | adult | 48 | 2015 | | *tetM* | | | x | | | ERR1742049 |
| PHEGBS0114 | VI | | | ST1 | blood | n/a | n/a | 2014 | | x | | | *mefA/msrD* | | | ERR1741639 |
| PHEGBS0297 | II | | | ST1 | blood | adult | 70 | 2014 | | *tetM, tetK* | | | *msrA* | | | ERR1741498 |
| PHEGBS0100 | II | | | ST3 | blood | adult | 49 | 2014 | | *tetW* | | | x | | | ERR1741744 |
| **Serotype Ia** | | | | | | | | | | | | | | | | |
| PHEGBS0770 | Ia | | | ST23 | blood | infant | 0 | 2015 | | *tetM* | | | *mefA/msrD* | | | ERR1742118 |
| PHEGBS0771 | Ia | | | ST23 | rectal swab | infant | 0 | 2015 | | *tetM* | | | *mefA/msrD* | | | ERR1741467 |
| PHEGBS0832 | Ia | | | ST23 | rectal swab | infant | 0 | 2015 | | *tetM* | | | *mefA/msrD* | | | ERR2320014 |
| PHEGBS0773 | Ia | | | ST23 | blood | infant | 0 | 2015 | | *tetM* | | | *mefA/msrD* | | | ERR1741975 |
| PHEGBS0831 | Ia | | | ST23 | blood | infant | 0 | 2015 | | *tetM* | | | *mefA/msrD* | | | ERR1924356 |
| PHEGBS0495 | Ia | | | ST23 | blood | adult | 94 | 2015 | | *tetM* | | | x | | | ERR1741920 |
| PHEGBS0503 | Ia | | | ST23 | blood | adult | 75 | 2015 | | *tetM* | | | x | | | ERR1741579 |
| PHEGBS0512 | Ia | | | ST23 | blood | adult | 32 | 2015 | | *tetM* | | | x | | | ERR1741958 |
| PHEGBS0514 | Ia | | | ST23 | blood | infant | 0 | 2015 | | *tetM* | | | x | | | ERR1742099 |
| PHEGBS0522 | Ia | | | ST23 | blood | infant | 0 | 2015 | | *tetM* | | | x | | | ERR1741962 |
| PHEGBS0525 | Ia | | | ST23 | blood | infant | 0 | 2015 | | *tetM* | | | x | | | ERR1741463 |
| PHEGBS0527 | Ia | | | ST23 | blood | adult | 26 | 2015 | | *tetM* | | | x | | | ERR1742059 |
| PHEGBS0542 | Ia | | | ST23 | blood | infant | 0 | 2015 | | *tetM* | | | x | | | ERR1742091 |
| PHEGBS0544 | Ia | | | ST23 | CSF | infant | 0 | 2015 | | x | | | x | | | ERR1742076 |
| PHEGBS0547 | Ia | | | ST23 | blood | adult | 88 | 2015 | | *tetM* | | | x | | | ERR1741621 |
| PHEGBS0589 | Ia | | | ST23 | blood | adult | 73 | 2015 | | *tetM* | | | x | | | ERR1741794 |
| PHEGBS0591 | Ia | | | ST23 | blood | infant | 0 | 2015 | | *tetM* | | | x | | | ERR1741921 |
| PHEGBS0611 | Ia | | | ST23 | blood | infant | 0 | 2015 | | *tetM* | | | x | | | ERR1741688 |
| PHEGBS0615 | Ia | | | ST23 | blood | adult | 33 | 2015 | | *tetM* | | | x | | | ERR1741741 |
| PHEGBS0621 | Ia | | | ST23 | blood | infant | 0 | 2015 | | *tetM* | | | *mefA/msrD* | | | ERR1741848 |
| PHEGBS0623 | Ia | | | ST23 | blood | adult | 67 | 2015 | | *tetM* | | | x | | | ERR1741456 |
| PHEGBS0625 | Ia | | | ST23 | blood | adult | 68 | 2015 | | *tetM* | | | *mefA/msrD* | | | ERR1742108 |
| PHEGBS0647 | Ia | | | ST23 | blood | infant | 0 | 2015 | | *tetM* | | | x | | | ERR1741420 |
| PHEGBS0651 | Ia | | | ST23 | blood | infant | 0 | 2015 | | *tetM* | | | x | | | ERR1741600 |
| PHEGBS0658 | Ia | | | ST23 | blood | adult | 82 | 2015 | | *tetM* | | | x | | | ERR1741901 |
| PHEGBS0673 | Ia | | | ST23 | blood | infant | 0 | 2015 | | *tetM, tetO* | | | *ermB, lnuB* | | | ERR1741807 |
| PHEGBS0674 | Ia | | | ST23 | blood | infant | 0 | 2015 | | *tetM* | | | x | | | ERR1742003 |
| PHEGBS0679 | Ia | | | ST23 | blood | infant | 0 | 2015 | | *tetM* | | | x | | | ERR1742052 |
| PHEGBS0686 | Ia | | | ST23 | blood | infant | 0 | 2015 | | *tetM* | | | x | | | ERR1741624 |
| PHEGBS0702 | Ia | | | ST23 | blood | infant | 0 | 2015 | | *tetM* | | | x | | | ERR1741757 |
| PHEGBS0714 | Ia | | | ST23 | blood | infant | 0 | 2015 | | x | | | x | | | ERR1742081 |
| PHEGBS0719 | Ia | | | ST23 | blood | infant | 0 | 2015 | | *tetM* | | | x | | | ERR1742106 |
| PHEGBS0748 | Ia | | | ST23 | blood | infant | 0 | 2015 | | *tetM* | | | x | | | ERR1741724 |
| PHEGBS0752 | Ia | | | ST23 | blood | infant | 0 | 2015 | | *tetM* | | | x | | | ERR1741884 |
| PHEGBS0758 | Ia | | | ST23 | blood | infant | 0 | 2015 | | *tetM* | | | x | | | ERR1741967 |
| PHEGBS0767 | Ia | | | ST23 | blood | infant | 0 | 2015 | | *tetM* | | | *mefA/msrD* | | | ERR1741874 |
| PHEGBS0769 | Ia | | | ST23 | blood | infant | 0 | 2015 | | *tetM* | | | x | | | ERR1741430 |

Abbreviations: CSF – cerebrospinal fluid; Abx – antibiotic; adult – aged 18 and over; infant – less than 1 year old.

**Suppl. Table 2. Phenotypic antimicrobial susceptibility characteristics of GBS serotype V cluster isolates.**

| Serotype V isolate | MLST | Antimicrobial susceptibility (MIC) | | | | | |
| --- | --- | --- | --- | --- | --- | --- | --- |
|  |  | **Penicillin** | **Clindamycin** | **Erythromycin** | **Tetracycline** | **Teicoplanin** | **Vancomycin** |
| **1** | ST 1 | <=0.060 | >8.0 | >16 | >8.0 | <=0.5 | <=0.5 |
| **2** | ST 1 | <=0.060 | >8.0 | >16 | >8.0 | <=0.5 | <=0.5 |
| **3** | ST 1 | <=0.060 | >8.0 | >16 | >8.0 | <=0.5 | <=0.5 |
| **4** | ST 1 | <=0.060 | >8.0 | >16 | >8.0 | <=0.5 | <=0.5 |

**Supplementary Figures**

**Suppl. Figure 1. Whole genome comparison of GBS serotype V outbreak strains and available serotype V reference sequences.** BRIG [8] was used to present BLASTN comparisons of the whole genome sequences of the outbreak strains and available reference sequences of GBS serotype V. The innermost ring shows genome scale in kilobase pairs and subsequent rings show BLASTN comparisons. Data for all three panels is the same: the innermost to outermost rings are outbreak strains: (1) PHEGBS0159 (2) PHEGBS0161 (3) PHEGBS0160, (4) PHEGBS0162; and reference sequences: (5) SS1 (NZ_CP010867.1), (6) 09mas018883 (NC_021485.1), and (7) 2603V/R (NC_004116.1) respectively. Panel A shows the BLASTN of strains PHEGBS0159 compared to other strains. The main difference is that all four outbreak strains have Tn917 (the most outer circle 8), carrying *ermB*, while reference sequences do not. Otherwise, reference sequences 09mas01883 and SS1 have very similar genomic contents to outbreak strains. Panel B shows reference SS1 compared to the outbreak strains and other reference sequences. The major difference between SS1 and outbreak strains is that mobile genetic element (MGE) RDF.1 is missing from the outbreak strains but is present in SS1 and 09mas018883. RDF.1 and RDF.2 difference between SS1 and 2603V/R were described in more detail by Flores *et al.*^[7]^ Panel C shows BLASTN comparison of reference sequence 09mas01883 to outbreak strains and other two reference sequences. The main differences between 09mas01883 and the outbreak strains are the MGE RDF.1 (outer ring 8) and major parts of lac.2 operon (outer ring 8); both these are lacking in the outbreak strains.

**Suppl. Figure 2. Phylogenetic analysis of outbreak GBS and other UK and North America GBS serotype V ST1 strains.** A phylogeny tree based on 2555 core SNPs identified between 22 serotype V ST1 UK GBS isolates and 40 serotype V ST1 isolates from N. America [7]. As before, serotype V ST1 outbreak isolates cluster together with one SNP difference between PHEGBS0161 and the other three outbreak isolates – PHEGBS0159, PHEGBS160 and PHEGBS162; these isolates are coloured in red. The closest North American isolate SRR1790780 was 55-56 SNPs away from the outbreak isolates, while the most distant North American isolate SRR1790793 was 332-333 SNPs away from the outbreak isolates. Branches coloured in blue indicate isolates that carried both tetracycline as well as macrolide/lincosamine resistance genes. Adult and neonatal case isolates were intermixed. All North American isolates were from adults, and UK isolates were from adults and neonates (see Suppl. Table 1). UK (PHEGBS) and North American isolates (SRR) were intermixed indicating that GBS strains are not limited to a geographical location (UK isolates are coloured in blue and North America isolates are in black).
